# DiffMC‐Gen: A Dual Denoising Diffusion Model for Multi‐Conditional Molecular Generation

**DOI:** 10.1002/advs.202417726

**Published:** 2025-04-01

**Authors:** Yuwei Yang, Shukai Gu, Bo Liu, Xiaoqing Gong, Ruiqiang Lu, Jiayue Qiu, Xiaojun Yao, Huanxiang Liu

**Affiliations:** ^1^ Faculty of Applied Sciences Macao Polytechnic University Macao 999078 China

**Keywords:** deep learning, diffusion model, drug design, molecular generation, multi‐objective optimization

## Abstract

The precise and efficient design of potential drug molecules with diverse physicochemical properties has long been a critical challenge. In recent years, the emergence of various deep learning‐based de novo molecular generation algorithms offered new directions to this issue, among which denoising diffusion models have demonstrated significant potential. However, previous methods often fail to simultaneously optimize multiple properties of candidate compounds, which may stem from directly employing nongeometric graph neural networks (GNNs), rendering them incapable of accurately capturing molecular topologic and geometric information. In this study, a dual denoising diffusion model is developed for multi‐conditional molecular generation (DiffMC‐Gen), which integrates both discrete and continuous features to enhance its ability to perceive 3D molecular structures. Additionally, it involves a multi‐objective optimization strategy to simultaneously optimize multiple properties of the target molecule, including binding affinity, drug‐likeness, synthesizability, and toxicity. From the perspectives of both 2D and 3D molecular generation, the molecules generated by DiffMC‐Gen exhibit state‐of‐the‐art (SOTA) performance in terms of novelty and uniqueness, meanwhile achieving comparable results to previous methods in drug‐likeness and synthesizability. Furthermore, the generated molecules have well‐predicted biological activity and druglike properties for three target proteins—LRRK2, HPK1, and GLP‐1 receptor, while also maintaining high standards of validity, uniqueness, and novelty. These results underscore its potential for practical applications in drug design.

## Graphical abstract

DiffMC‐Gen, a dual‐diffusion model for 2D and 3D molecular generation, simultaneously optimizes multiple key objectives across the drug design process, enabling the generation of novel, target‐specific small‐molecule ligands with high therapeutic potential.

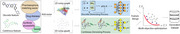

## 1 Introduction

Drug discovery is a highly complex process characterized by long timelines, high risks, and substantial investments. In recent years, computer‐aided drug design (CADD) has shown great potential in assisting various stages of drug development, significantly improving the efficiency of drug discovery.^[^ ^1^ ^]^ However, the vast potential chemical space of candidate drug molecules, estimated to be as large as 10^60^, presents a significant challenge. Identifying potential drug compounds from such an immense chemical space is akin to searching for a needle in a haystack. In addition, it involves simultaneously refining various properties of the target compound which are essential for further research, such as novelty, uniqueness, drug‐likeness, and synthesizability. Therefore, overcoming numerous obstacles, especially multi‐objective optimization, is critical for drug discovery. Experimental approaches often focus on one or a few highly correlated properties at a time, while requiring substantial time investment. With the rapid advancement of artificial intelligence, deep learning‐based molecular generation models have emerged, offering new possibilities to address this challenge.

The 3D geometry of a molecule is a key determinant of its physicochemical properties, which directly impact its pharmacology, pharmacokinetics, metabolism, and toxicity. Consequently, exploring molecular structures in 3D space is advantageous for advancing drug discovery. Graph neural networks (GNNs) excel at capturing the explicit characteristics of molecular graphs, making them well‐suited for learning structural information. GNNs integrate multiple algorithms dedicated to generating the molecules in the 3D space, such as autoregressive models, latent representation‐based models, equivariant normalizing flows, and diffusion models. Among these methods, diffusion models can more effectively capture the intrinsic features of the data through a series of diffusion steps, thereby generating molecular graphs with greater realism and detail.^[^ ^2^ ^]^ This characteristic facilitates a deeper understanding of the process and mechanisms of molecular generation, which arouses the interest of researchers.^[^ ^3^ ^]^ For 3D molecular generation based on diffusion models, a common approach involves separately addressing chemical bonds and intramolecular weak interactions by introducing noise to the adjacency matrix of edges.^[^ ^4^ ^]^ Discrete molecular graphs are embedded into continuous space for processing, making crucial structural information undefined or obscured. This operation also hinders accurately capturing the relationships between atoms and bonds in the original molecular graph, leading to false connecting.^[^ ^4^, ^5^ ^]^

A valuable strategy for addressing the above issue in the diffusion model for 3D molecular generation is to integrate discrete and continuous diffusion models, enabling the management of both 2D and 3D features to guide the bond formation process.^[^ ^6^ ^]^ Although these methods improve molecular stability, their conditional generative capacity remains limited to a narrow range of heavy atom sets. Besides, current generative models based on diffusion models are barely concerned with the special properties of drug candidates, leading to a disconnect between evaluation metrics and real‐world performance. Therefore, there is an urgent need to make advancements in model design, particularly focusing on enhancing accuracy, scalability, and the capacity to create genuinely innovative and varied chemical compounds that fulfill the intricate demands of drug discovery.^[^ ^7^ ^]^

In this paper, we proposed a novel method named DiffMC‐Gen (dual denoising Diffusion model for Multi‐Conditional molecular Generation) by integrating the discrete and continuous denoising diffusion model to address these challenges. We incorporate the Dynamically Composable Multi‐Head Attention method into the discrete graph network architecture, which reduces network parameters and improves operational speed.^[^ ^8^ ^]^ Furthermore, we introduce a novel local hierarchy of 3D isomorphism encoding algorithm for capturing geometric features, especially molecular special information.^[^ ^9^ ^]^ This methodology improves traditional equivariant processing by incorporating considerations for bond angles and employing spatial distance transformations to establish bond connections. Additionally, DiffMC‐Gen simultaneously optimizes pharmacophore matching coefficients,^[^ ^10^ ^]^ acute toxicity evaluations, as well as Quantitative Estimate of Drug‐likeness (QED)^[^ ^11^ ^]^ and Synthetic accessibility (SA)^[^ ^12^ ^]^ scores to enhance drug‐likeness and potential biological activity. Finally, we demonstrate the utility of DiffMC‐Gen through case studies focusing on Hematopoietic progenitor kinase 1 (HPK1), Leucine‐rich repeat kinase 2 (LRRK2), and Glucagon‐like peptide‐1 (GLP‐1) receptor. Evaluation results show that the generated molecules based on DiffMC‐Gen align well with specified pharmacophore hypotheses and pharmacokinetic criteria while maintaining high validity, uniqueness, and novelty.

## 2 Methods

In this section, we will provide a brief overview of the datasets used, along with a detailed description of the design concepts and technological advancements incorporated into our model.

### 2.1 Datasets

For the development and assessment of the molecular generation model, three datasets were used, including Quantum Machine 9 (QM9),^[^ ^13^ ^]^ Cambridge Structural Database (CSD) from Cambridge Crystallographic Data Centre (CCDC), and Molecular Sets (MOSES).^[^ ^14^ ^]^ Among the three datasets, QM9 and MOSES are the most commonly used and widely recognized datasets in the development of molecular generation methods. In addition, the Cambridge Structural Database (CSD), which provides reliable 3D geometric structures for a wide range of compounds, is also utilized in our models to enhance the reliability of the generated 3D molecular conformations. The structural information statistics of the molecules in the three datasets are provided in Table S1 (Supporting Information). A detailed introduction to the three datasets is given below.

#### 2.1.1 QM9

The QM9 dataset consists of computed geometric, energetic, electronic, and thermodynamic properties for 134k stable small organic molecules made up of C, H, O, N, and F. The molecules within the QM9 dataset typically consist of nine heavy atoms without hydrogen atoms, and generally, 18 atoms when hydrogen atoms are included. This dataset plays an important role in evaluating the capability of generating small, diverse, and physically realistic molecules with defined quantum properties.

#### 2.1.2 CSD

The CSD dataset essentially includes all published organic and metal‐organic compounds with crystal structures. This dataset contains over 60K molecules with experimental 3D conformations. Molecules that deviate significantly from drug‐like structures or contain more than 40 atoms have been filtered out. Since stable crystal conformations serve as a reliable reference for small‐molecule generation, leveraging this dataset may improve the model's ability to generate stable 3D molecular conformations.

#### 2.1.3 MOSES

This dataset consists of 1.9 million clean, lead‐like molecules sourced from the ZINC database. Each molecule contains an average of 29 atoms. This dataset designed to encompass drug‐like and bioactive molecules, provides a robust benchmark for assessing the capacity to generate synthetically accessible and drug‐like compounds. This dataset does not include 3D structure information originally. To train our model to generate 3D molecular structure, we need to generate 3D conformers for each compound in this dataset. As we know, 3D conformers play an important role in the development of the 3D molecular generation method. The commonly used methods for 3D conformation generation mainly include rule‐based methods, distance geometry, systematic search, stochastic methods, molecular dynamics, deep learning‐based methods, and hybrid methods.^[^ ^15^ ^]^ Hybrid methods and deep learning‐based approaches are increasingly popular due to their ability to combine speed, accuracy, and scalability. RDKit integrates rule‐based and stochastic methods to generate molecular 3D conformers, which belong to hybrid methods for 3D conformation generation. It has advantages in balancing speed, accuracy, and diversity for 3D conformation generation. Thus here, we used RDKit^[^ ^16^ ^]^ to generate 3D structures and employed a Universal Force Field (UFF)^[^ ^17^ ^]^ for minimum energy optimization.

To test our molecular generation model, several targets including HPK1, LRRK2, and GLP1 were used. Their 3D structures including HPK1 (PDB ID: 7M0M), LRRK2 (PDB ID: 8FO7), and GLP‐1 receptor (PDB ID: 7S15) were downloaded from the PDB database.^[^ ^18^ ^]^ The detailed medicinal therapeutic significance of the three targets can be found in Supporting Information.

### 2.2 Model Design

**Figure 1:**
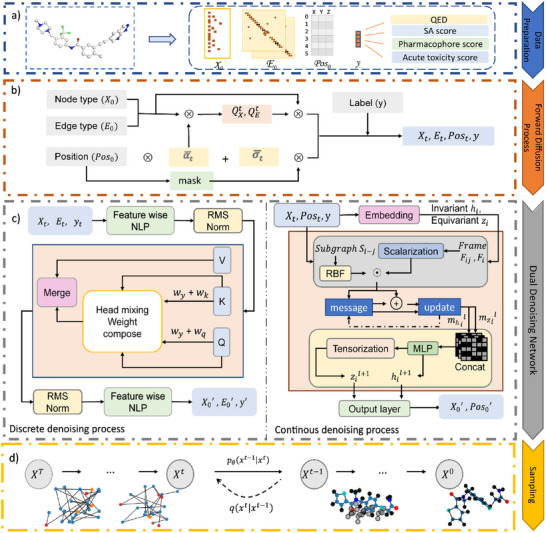
The overall architecture of DiffMC‐Gen. The model is composed of four components: a) data preparation, b) the noise diffusion process, c) the denoising diffusion process for both kinds of graphs, and d) constrained sampling.

Diffusion models have been applied successfully to image generation and related fields. It defines a process that gradually introduces noise into the data and then trains neural networks to reverse this degradation through iterative denoising. As shown in **Figure** **1**a, the inputs of DiffMC‐Gen include discrete molecular features, global features, and conformational geometrics. Discrete graphs are decomposed into categorical node and edge attributes, which are represented by one‐hot encodings and denoted by the space *X* and *E*, respectively. The global feature of each graph is concentrated on the feature y, which involves structural features (e.g., cycle features and spectral features) and molecular features (e.g., charge, valency, and weight distributions). Multiple constrained properties are incorporated with dynamic weight w. The conformational data (positions, distances, angles) are also encoded as part of a continuous graph. Categorical and geometric features are transformed into discrete and continuous representations, respectively.

Figure 1b shows the gradual addition of noise leads to *x*_0_ losing its distinguishable features progressively and changing to q(*x_t_* |*x*_*t* − 1_) in the forward diffusion process. For discrete graphs, noise is represented by transition matrices  *Q*1,…,*QT*, which influence the marginal distribution of node and edge types. For continuous graph, Gaussian noise is applied to the atomic 3D coordinates and atomic weights over *T* sampling steps, resulting in outcomes that follow an isotropic Gaussian distribution $N\left(x_{t}\left|\alpha_{t}x_{\left(t-1\right)}\right.,\sigma_{t}^{2}I\right)$. Here, α_*t*_ controls the amount of signal retained, and σ_*t*_ controls the amount of noise added. The noisy data are tokens as inputs of discrete and continuous denoising networks. Denoising diffusion networks learn to inert the diffusion trajectories to predict the clean output *x*′ from *x_t_*, which need to learn the distribution: *p*_θ_(*x*_*t* − 1_|*x_t_*). Then the Kullback–Leibler divergence between *x*′ and *x*_0_ would be minimized to approximate the conditioned probability distributions.

#### 2.2.1 Discrete Denoising Generative Network: Graph Transformer with Dynamically Composable Multihead Attention

In this study, we employ graph transformer layers to construct the discrete graph denoising network. The graph transformer is inspired by the success of transformer in natural language processing (NLP) and computer vision, combined with the proven effectiveness of graph neural networks (GNNs).^[^ ^19^ ^]^ Through the integration of graph inductive bias, graph transformers adjust to dynamic and heterogeneous graphs, utilizing feature equivalence across nodes and edges. Nevertheless, the computational expenses and memory usage increase significantly when aiming to improve the scalability and efficiency of applying self‐attention to large‐scale graphs, like those in datasets such as MOSES and CSD.

Graph attention mechanisms enable transformers to prioritize and emphasize the most relevant and important elements for the given task. Dynamically Composable Multi‐Head Attention (DCMHA) integrates edge attention scores and conditional features as weight matrices, enhancing its ability to explore druglike molecules under multiple condition constraints while optimizing computational efficiency.^[^ ^8^ ^]^ Figure 1c illustrates how the approach facilitates information exchange between different attention heads and guides the extraction of edge features from the latent space. Additionally, it helps to mitigate the issue of insufficient computational resources. The Adaptive Instance Norm layer is also used to integrate conditional information with node and edge information. Training on nodes, edges, and weighted global features helps prevent DiffMC‐Gen from over‐relying on conditional information.

#### 2.2.2 Continuous Denoising Generative Network: Powerful and Efficient Geometric Graph Neural Networks

Accurately incorporating local conformation information is crucial for the 3D generation of small molecules. Currently, Equivariant Graph Neural Network (EGNN)^[^ ^9^ ^]^ and other methods that widely adopted in denoising diffusion framework for 3D molecular generation. When they capture local geometry and global topological structures at multiple scales, they rely on node coordinate updates, which leads to high memory and computational requirements.^[^ ^4^, ^5^, ^20^ ^]^ Partial information may be lost when the topological structure of molecules is complex or when a molecule possesses chiral characteristics.^[^ ^21^ ^]^ In this work, we employed SE(3)‐equivariant local isomorphism to quantify the local similarity of 3D structures.^[^ ^9^ ^]^ This powerful and efficient geometric graph neural network is constructed as a continuous denoising generative network.

The denoising diffusion process has incorporated Markov chains $\sigma_{1}^{2},\sigma_{2}^{2},\text{…},\sigma_{T}^{2}$ to denoise molecular graph inputs *G*, which are associated with atomic weights and positions  $x_{i}\in R^{3}$ of each node. A local frame transition block here is designed to elucidate global alterations among local geometries. While maintaining equivariance, it enhances sensitivity to local geometric structures. Leveraging implicit geometry‐aware mechanisms avoids direct coordinate updates and efficiently extracts geometric information through edge weights and attention mechanisms. The multi‐level attention framework also effectively captures both local geometric details and global topological features. Meanwhile, the pharmacophore matching coefficient is incorporated as conditional guidance, steering the geometric denoising process. This strategy aims to improve the potential bioactivity of the generated 3D molecular structures to their target molecules.

To analyze the impact of key components in DiffMC‐Gen, ablation experiments were performed and the detailed process together with results were given in the supporting information.

#### 2.2.3 Constrained Properties

The core of drug discovery lies in the creation of novel molecules with the desired properties. Thus here, to ensure the generated molecules with the desired properties, various drug‐related properties including activity, drug‐likeness, synthetic accessibility, and toxicity are used as constraints during the generation process. Drug‐likeness is influenced by multiple factors, including physicochemical properties, and pharmacokinetics. The interaction among these factors is often more critical than any single attribute,^[^ ^22^ ^]^ which also suggests that molecular generation is a multi‐objective optimization problem. In this study, a set of comprehensive evaluation metrics are employed as constrained indicators to avoid unintended biases from multiple simple criteria. The properties used are described below.

*Pharmacophore matching score*. To improve the potential activity of generated molecules, a ligand‐based pharmacophore matching score is commonly used as one of the optimization objectives to guide the generation of highly active molecules.^[^ ^10^, ^23^ ^]^ In this work, we downloaded the target‐related inhibitors or agonists from the PubChem database to construct a proper pharmacophore hypothesis. The selected active molecules for specific targets are classified into five distinct categories based on their core scaffolds. Common functional moieties were utilized to construct a pharmacophore model for each target. Then the spatial distribution of pharmacophore sites and the pharmacophoric features of the reference dataset were represented as a complete graph.^[^ ^10^ ^]^ Details of the process are shown in Figure S1 (Supporting Information). The highest graph matching scores to the pharmacophore hypothesis are set as a constrained indicator, which ranges from 0 (unfavorable) to 1 (favorable).

*QED*. Drug‐likeness is an important criterion used to assess the potential of compounds for drug development, which is significant in increasing the success rate of drug development and reducing costs. Quantitative estimates of drug‐likeness (QED) address the limitations of drug‐likeness rules based on physical and chemical properties. It integrates eight physicochemical properties (molecular weight, H‐bond donors, H‐bond acceptors, LogP, charge, aromaticity, solubility, and stereochemistry) to produce a score ranging from 0 to 1. A score closer to 1 indicates a more drug‐like molecule. QED can serve as an optimization target or assessment metric, guiding the model to generate compounds with high drug‐likeness.^[^ ^11^ ^]^

*SA score*. The essence of drug discovery is the design of novel molecules that achieve a balance between efficacy, safety, and manufacturability. The synthetic accessibility of generated compounds is the main concern for medicinal chemists. The synthetic accessibility score (SA score) is calculated by integrating fragment contributions and a complexity penalty in a predefined database. Fragment contributions have been derived from the analysis of one million representative molecules in PubChem, where frequent fragments receive positive scores and less frequent ones receive negative scores. On the other hand, the molecular complexity penalty reflects the presence of non‐standard structural features, including spiro rings, ring fusions, multiple potential stereocenters, and rings of size > 8. SA score is normalized within the range from 0 (favorable) to 1 (unfavorable). It is calculated sufficiently fast and provides results consistent with an estimation of ease of synthesis by experienced medicinal chemists. It is widely used as a constrained objective to assess the synthesized difficulty of the generated compounds.^[^ ^12^ ^]^

*Acute Oral Toxicity*. Toxicity is one of the major factors contributing to the failure of drug development. Here, we used acute oral toxicity as one constrained objective to reduce the toxicity of the generated compounds. Acute oral toxicity is the toxic reaction of a drug over a certain period after multiple administration within a single or 24‐hour period. As shown in the PubChem database, Rat oral LD50 is always used as the evaluation criterion for the acute toxicity of molecules. The normalized acute toxicity dataset with the Lethal Dose Fifty (LD50) values was downloaded from the TOXRIC database.^[^ ^24^ ^]^ We developed a machine learning prediction model based on this dataset to predict the likelihood of acute toxicity according to the 2D structure of the compounds. The detailed performance of this prediction model is shown in Figures S2 and S3 (Supporting Information). The value of toxicity is normalized within the range from 0 (favorable) to 1 (unfavorable).

#### 2.2.4 Multiobjective Optimization Strategy

In this work, generated molecules would be optimized in the generated process by multiple comprehensive evaluated metrics to enhance their potential. Here, we developed a multi‐objective optimization strategy that alternates between a conditional diffusion model and an unconditional diffusion model to enhance control over the generated outputs. The conditional diffusion model incorporates both discrete and continuous diffusion processes, while the unconditional model focuses solely on discrete diffusion. In the conditional diffusion model, the discrete sub‐model explicitly embeds the conditional distribution, which incorporates four different constraints, while employing an attention mechanism for implicit control. Meanwhile, the continuous sub‐model only considers the pharmacophore matching coefficient. The unconditional diffusion model training focuses on discrete sub‐models without incorporating global features. A regressor‐guided diffusion approach is employed in sampling, where ϕ_θ_ Modulates the predicted distribution at each sampling step, steering it toward molecular graphs with the desired properties.

#### 2.2.5 Evaluation Metrics

We adopt widely used metrics to evaluate the quality of molecules generated by our model. 1) *Glide score (Gscore)* estimates the binding affinity between ligand and target. We assessed different docking protocols on the selected targets in the case study and ultimately selected the Glide module of Schrödinger to evaluate the binding affinity and binding pose of representative molecules due to its superior performance in molecular docking verified by many researchers ^[^ ^25^ ^]^ and our evaluation results. The proteins and molecules were first prepared by using the *Protein Preparation Wizard* and *LigPrep* modules in Schrödinger, respectively, with all the default settings.^[^ ^26^ ^]^ Then Glide SP was employed with semi‐flexible receptor docking, generating 20 poses per ligand without specific constraints. 2) *Success rate* is defined as the proportion of generated molecules that exhibit desirable drug‐like properties, specifically those with QED ≥ 0.6 and SA score ≤ 0.4, indicating high drug‐likeness and favorable synthetic accessibility. 3) *Novelty* represents the ability of the model to generate molecules that are structurally different from those in the training set or existing known molecules. 4) *Uniqueness* is judged by assessing whether the chemical graph of a generated molecule is unique among the produced samples. 5) *Validity* is the percentage of generated molecules that obey common chemical rules (like valency). 6) *Diversity* comprises two attributes: *Internal Diversity (IntDiv)*, which assesses the variety within the generated set by uncovering structural distinctions among the generated molecules, and *the Fréchet ChemNet Distance (FCD)*, which gauges the resemblance between the distributions of generated and real molecules, encompassing both structural and chemical similarities.^[^ ^27^ ^]^ 7) *The energy ratio* in PoseBusters is used to evaluate the reliability of generated molecules.^[^ ^28^ ^]^ This metric provides a more reliable assessment of molecular feasibility by comparing the stability of the generated conformation against a distribution of unconstrained conformations. It represents the fraction of molecules in the generated dataset whose calculated energy does not exceed seven times the average energy of an ensemble of 50 unconstrained conformations generated for each molecule.

#### 2.2.6 Statistical Analysis

*Preprocessing of data*: RDKit was employed to standardize molecules across all datasets, ensuring the removal of incorrectly processed molecules and performing deduplication. Additionally, the constrained properties of molecules were normalized to ensure data consistency.

*Data presentation*: All experiments were performed in three replicates. The comparison results between different methods are reported as the mean values of the evaluation metrics.

*Sample size for each statistical analysis*: The sample size of each statistical analysis corresponds to the number of generated or randomly selected molecules in each experiment. Specific sample sizes are provided in relevant figure legends and table statements.

*Statistical methods*: In this study, the evaluation primarily relies on predictive estimations and proportion‐based comparisons. No inferential statistical tests (e.g., hypothesis testing) were applied, as the focus is on performance assessment using predefined evaluation metrics.

*Software for statistical analysis*: All statistical analyses were conducted in Python 3.8.

## 3 Results and Discussion

### 3.1 The Quality of Generated Molecules from DiffMC‐Gen

#### 3.1.1 The Performance Evaluation of DiffMC‐Gen for 2D Molecular Generation

For the task of 2D molecular generation, we compared the generation performance between our model and several recently developed generation models with good performance. Among the selected models, MARS is a powerful and versatile molecular generation model by combines GNNs and the Markov chain Monte Carlo sampling method.^[^ ^29^ ^]^ LSTM with Hill Climbing (LSTM‐HC), a representative method in the GuacaMol benchmark, excels at generating molecules with nearly 90 nodes.^[^ ^30^ ^]^ JTVAE‐BO was designed to generate target‐oriented molecular graphs, effectively guiding the optimization of molecular structures.^[^ ^12^, ^31^ ^]^ DiGress is a discrete diffusion model, leveraging the exceptional denoising capabilities of diffusion models to learn the attributes of nodes and edges in molecular graphs. This model has achieved outstanding performance on molecular and graph datasets.^[^ ^32^ ^]^ GDSS constructed a graph diffusion process that models the joint distribution of the nodes and edges through a system of stochastic differential equations (SDEs).^[^ ^33^ ^]^ Graph DiT is built upon the exceptional combination of transformer and diffusion models and has been modified specifically for conditional molecular generation.^[^ ^34^ ^]^

Here, the comparison between DiffMC‐Gen and these baseline models was performed based on both the QM9 dataset and the MOSES dataset. On the QM9 dataset, DiffMC‐Gen was compared with baseline models, emphasizing the generation of small‐scale molecular graphs under the guidance of quantum chemical properties. Meanwhile, the MOSES dataset was used to assess its ability to produce a large volume of molecular graphs. **Table** **1** provides a summary of the overall assessment and comparison results.

**Table 1:** The evaluation and comparison of DiffMC‐Gen for 2D molecular generation (*n* = 2000).

Dataset	Model	Validity	Diversity		Novelty	Uniqueness	Success Rate
			IntDiv	FCD			
QM9	MARS^[^ ^29^ ^]^	**100.00%**	0.92	7.343	70.95%	22.19%	–
	LSTM‐HC^[^ ^30^ ^]^	**100.00%**	0.80	18.43	100.00%	100.00%	–
	DiGress^[^ ^32^ ^]^	99.39%	0.92	**0.90**	32.08%	96.16%	–
	GDSS^[^ ^33^ ^]^	95.13%	0.92	2.54	81.76%	97.74%	–
	Graph DiT^[^ ^34^ ^]^	92.21%	0.92	1.43	71.56%	88.54%	–
	**DiffMC‐Gen**	89.00%	**0.92**	2.57	**100.00%**	**100.00%**	–
MOSES	MARS^[^ ^29^ ^]^	99.8%	0.86	18.73	99.95%	35.12%	54.61%
	LSTM‐HC^[^ ^30^ ^]^	50.04%	0.86	9.97	3.67%	100.00%	85.04%
	JTVAE‐BO^[^ ^31^ ^]^	**100.00%**	0.87	17.06	100.00%	58.90%	51.70%
	DiGress^[^ ^32^ ^]^	86.34%	0.85	**0.81**	95.84%	100.00%	94.38%
	GDSS^[^ ^33^ ^]^	97.01%	0.89	17.96	100.00%	99.64%	28.07%
	Graph DiT^[^ ^34^ ^]^	76.48%	0.86	1.81	95.22%	98.90%	90.16%
	**DiffMC‐Gen**	81.39%	**0.89**	16.06	**100.00%**	**100.00%**	**95.23%**

From Table 1, it can be seen that DiffMC‐Gen generates molecules with good Validity, highest Uniqueness, and Novelty on both the QM9 and MOSES datasets. These indicators reflect that our model can effectively reconstruct topological information to generate molecules with simple or complex structures. Additionally, DiffMC‐Gen performs well on diverse metrics. The internal diversity of DiffMC‐Gen is 0.92 when trained on the QM9 dataset and 0.89 when trained on the MOSES dataset, demonstrating performance comparable to most baseline models and DiffMC‐Gen is good at discovering more diverse molecular structures even constrained by multiple indicators. Molecules generated by DiffMC‐Gen exhibit high FCD values, indicating that DiffMC‐Gen has greater potential to effectively explore the marginal distribution of node and edge types under multiple conditional constraints. Furthermore, the highest success rate, calculated based on a combination of QED and SA Score, highlights the effectiveness of the multi‐objective optimization strategy in our model.

#### 3.1.2 The Performance Evaluation of DiffMC‐Gen for 3D Molecular Generation

Two datasets are used to evaluate the performance of DiffMC‐Gen in generating novel 3D molecules with multi‐objective optimization, including the QM9 dataset for generating small‐scale molecular graphs and the CSD dataset for generating larger, drug‐like molecules. Baseline models selected for comparison with our model mainly include recent proposed models based on diffusion models. MDM is a dual diffusion‐based generative model designed to independently consider chemical bonds and intramolecular weak interactions.^[^ ^4c^ ^]^ GeoLDM integrates equivariant latent features into the latent space, enhancing the validity percentage of large molecules and the ability for controllable generation.^[^ ^35^ ^]^ GFMDiff reinforces the multi‐body relationship between binary edges and molecular geometry, effectively enhancing molecular stability as well as specific molecular properties.^[^ ^20^ ^]^ GCDM achieves enhanced Vina scores and high validity by prioritizing molecular stability in its design.^[^ ^5a^ ^]^

For a standardized assessment of 3D molecular generation performance, 2000 molecules generated from each model mentioned above were utilized. The properties optimized during training on the QM9 dataset align with those used in the 2D molecular generation task, whereas the properties optimized during training on the CSD dataset match those employed in the MOSES training process. Generally, the generation performance of the model will decline when the balance between global features and local features is not optimal. DiffMC‐Gen tries to overcome this problem.

As shown in **Table** **2**, DiffMC‐Gen shows excellent performance in both datasets QM9 and CSD. Especially, DiffMC‐Gen has the best Uniqueness and best or second‐best Validity compared with the baseline models. The novelty of generated molecules by DiffMC‐Gen is also prominent, with the best and second‐best results for CSD and QM9 datasets, respectively. This indicates that DiffMC‐Gen is capable of generating molecules distinct from those in the training dataset, even when the input information is complex. Meanwhile, the high validity indicates that DiffMC‐Gen can effectively learn the structure distribution of the training set. Here, the Energy ratio was used to evaluate the physicochemical plausibility of generated molecules. As shown in Table 2, DiffMC‐Gen demonstrates exceptional performance in the energy ratio metric, highlighting its capability to produce molecules with reasonable conformations. These results highlight DiffMC‐Gen's superior performance in 3D molecular generation tasks, showcasing its capability to efficiently balance multiple constraints.

**Table 2:** The evaluation and comparison of DiffMC‐Gen for 3D molecular generation (*n* = 2000).

Dataset	Methods	Validity	Uniqueness	Novelty	Energy Ratio
QM9	GFMDiff^[^ ^20^ ^]^	91.67%	100.0%	90.98%	–
	MDM^[^ ^4c^ ^]^	65.01%	44.18%	**99.93%**	7.00%
	GCDM^[^ ^5a^ ^]^	**92.70%**	93.40%	58.70%	**72.88%**
	GeoLDM^[^ ^35^ ^]^	76.60%	99.86%	74.90%	31.90%
	**DiffMC‐Gen**	89.00%	**100.00%**	93.33%	55.30%
CSD	GFMDiff^[^ ^20^ ^]^	53.20%	–	–	–
	MDM^[^ ^4c^ ^]^	25.85%	71.49%	98.38%	6.23%
	GCDM^[^ ^5a^ ^]^	46.8%	95.5%	95.5%	**12.25%**
	GeoLDM^[^ ^35^ ^]^	1.2%	81.82%	100.0%	5.77%
	**DiffMC‐Gen**	**75.00%**	**100.0%**	**100.0%**	11.35%

#### 3.1.3 Molecular Properties Distribution

**Figure 2:**
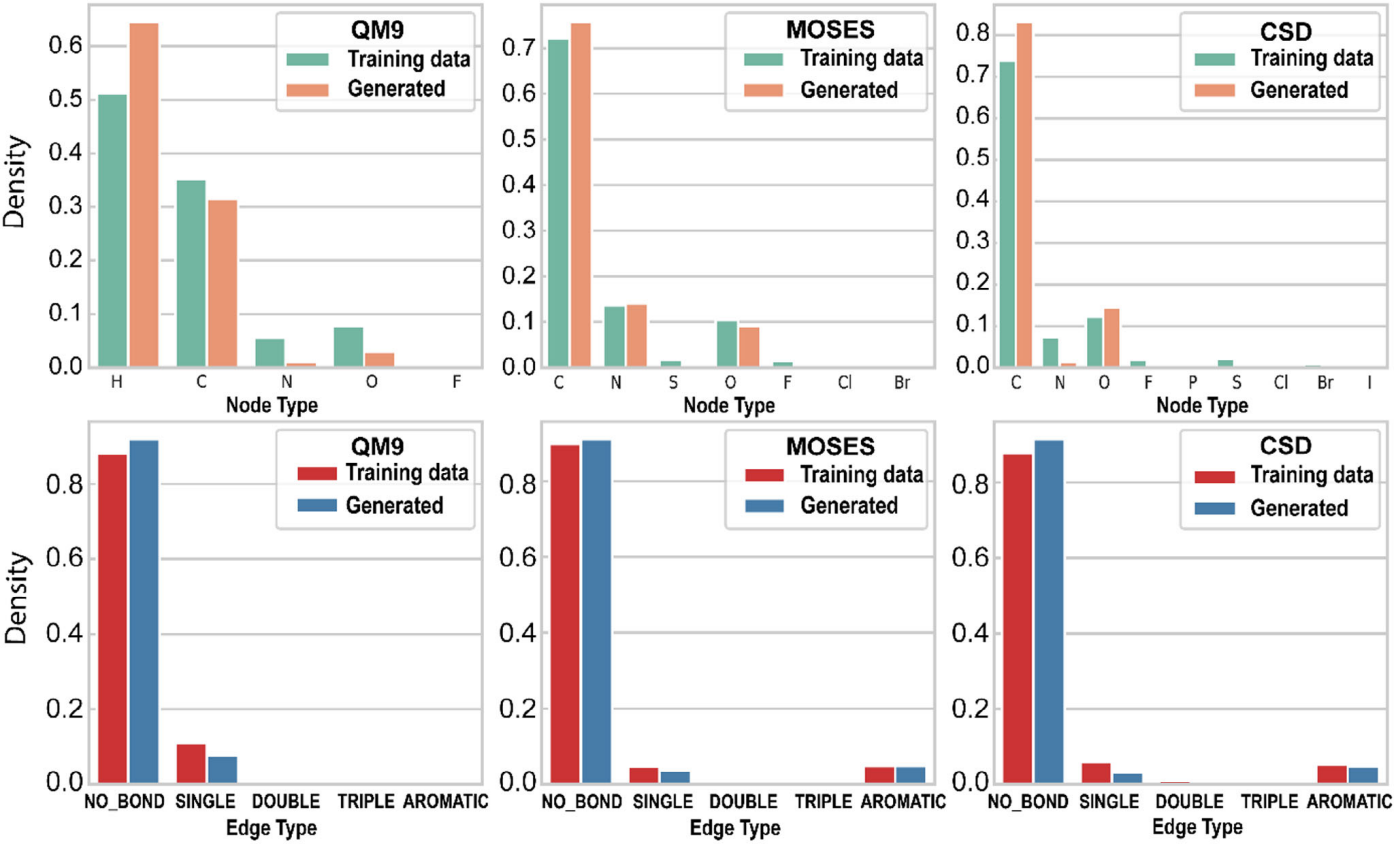
Histogram of distribution for atom and bond types of generated molecules (*n* = 10000) by DiffMC‐Gen and those in the training dataset.

We assessed the node type distribution and edge type distribution of molecules generated by DiffMC‐Gen and the molecules in the training set. As shown in **Figure** **2**, the atom type and bond type distributions of molecules generated by DiffMC‐Gen closely align with those of the training data, demonstrating its strong ability to learn molecular topological structure distributions. Based on the Similarity Property Principle,^[^ ^36^ ^]^ which posits that chemically similar molecules are likely to exhibit similar biological activities or properties, DiffMC‐Gen is expected to generate molecules with physicochemical and pharmacokinetic profiles that closely resemble those in the training set. To verify this, we further compared the molecules generated by DiffMC‐Gen with those from baseline models across four critical properties essential for drug development.

**Figure 3:**
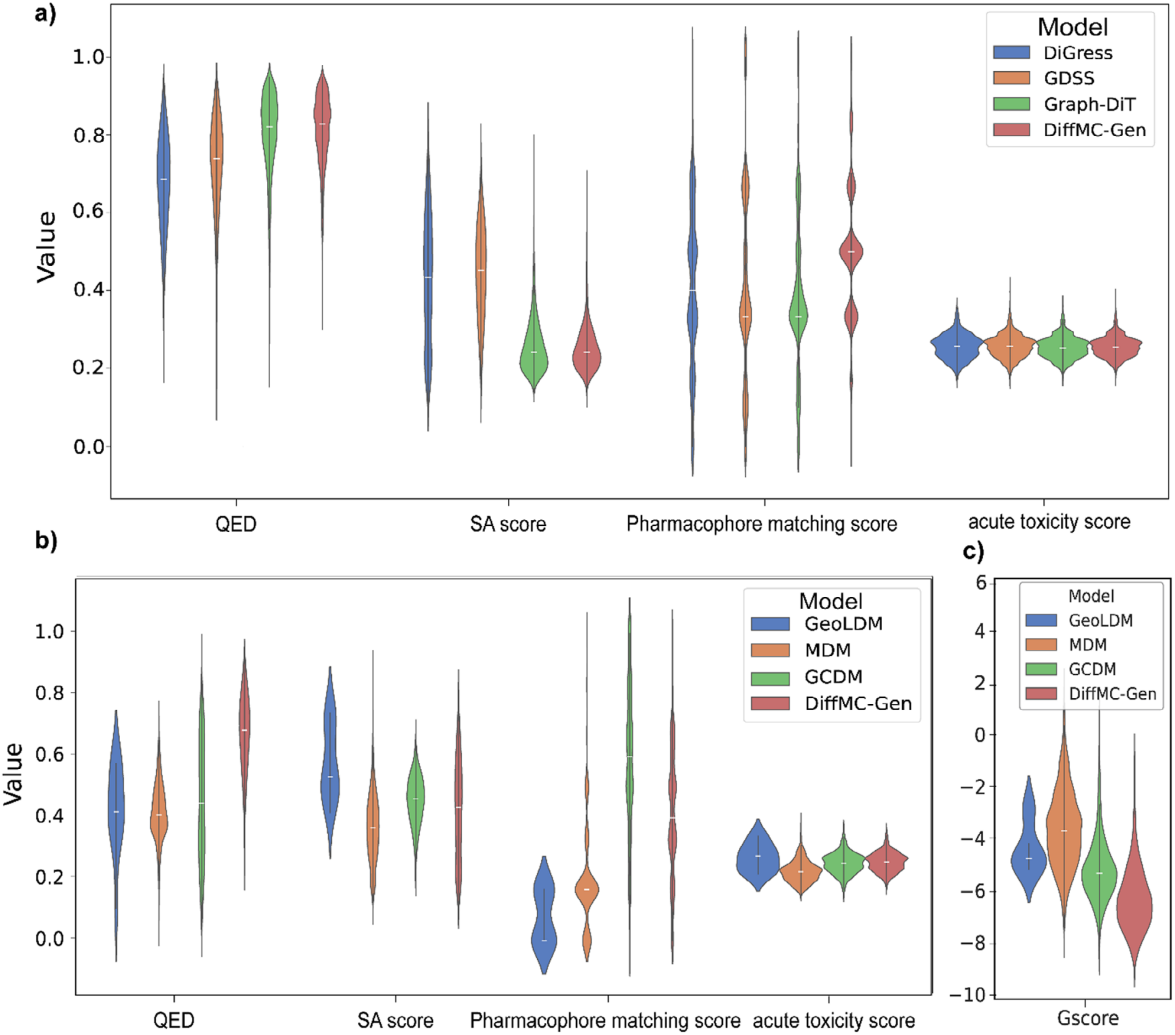
Comparison of the properties of generated molecules by DiffMC‐Gen and baseline methods for a) multi‐conditional 2D molecular generation models, b) multi‐conditional 3D molecular generation models, and c) the distribution of docking score in generated molecules (*n* = 1000).

We randomly selected 1000 molecules from the generated dataset in the 2D multi‐conditional molecular generation task to evaluate their properties and compare the distribution of these properties with those of molecules generated by the baseline models. The comparison results are given in **Figure** **3**a. As shown in Figure 3a, DiffMC‐Gen shows a similar drug‐likeness estimated score distribution to Graph‐DiT with the highest concentration around 0.8, slightly higher than DiGress and GDSS, indicating that DiffMC‐Gen has the substructure learning ability to reconstruct molecules that satisfy Lipinski's five rules. For synthetic accessibility, DiffMC‐Gen demonstrates comparable performance to Graph‐DiT, with both models achieving scores centered around 0.2, which is significantly better than those of DiGress and GDSS. This observation indicates that after incorporating multiple global features as constraints, DiffMC‐Gen can effectively adjust the structural complexity of the generated molecules and balance various constraint attributes. Instead, it can effectively balance the learning of both global and local features. Furthermore, the elevated distribution of the median pharmacophore matching coefficient in molecules generated by DiffMC‐Gen suggests that this model is particularly adept at identifying regions of chemical space enriched with target‐specific active structures, while simultaneously preserving favorable QED and SA scores. For potential toxicity, both DiffMC‐Gen and the baseline models exhibit a low probability of acute toxicity in the generated datasets. This is because acute toxicity is typically associated with specific representative atom types and structures, and the data used for the training set has undergone partial filtering. As a result, the models effectively learn and reproduce this broadly distributed property. From the above comparison, DiffMC‐Gen shows the potential to generate novel molecules with constrained properties.

As for the 3D molecular generation task, can our model generate molecules with the expected properties and reasonable 3D conformations? To answer this question, we further compared the distribution of expected properties of generated molecules by our model DiffMC‐Gen and other baseline models at multi‐conditional 3D molecular generative tasks. As illustrated in Figure 3b, DiffMC‐Gen demonstrates exceptional performance in generating molecules with strong drug‐likeness, as the majority of scores fall within the 0.7–0.8 range. DiffMC‐Gen also outperforms GeoLDM and MDM in pharmacophore matching, with the highest proportion of scores between 0.5 and 1.0, demonstrating the model's strength in leveraging conditional guidance during conformation generation. In terms of acute toxicity and synthetic accessibility distribution, DiffMC‐Gen performs similarly to baseline models, with most values concentrated in the desired attribute ranges. It shows that our model can still better adjust the structure complexity and the frequency of special structures after the optimization of geometric information.

Furthermore, we applied molecular docking to assess the potential binding ability of generated molecules to target. LRRK2 (PDB ID: 8FO7) was selected as a target to test our model and baseline models. As shown in Figure 3c, DiffMC‐Gen exhibits a Glide Gscore most frequently around −6.4, which is superior to all baseline models. This demonstrates that DiffMC‐Gen improves the potential activity of generated molecules by leveraging conditional 3D geometric optimization, underscoring its ability to design molecular structures with high bioactivity. These results further support that DiffMC‐Gen generates molecular structures with expected properties, especially good drug‐like properties and activity.

### 3.2 DiffMC‐Gen Can Generate Bioactive Molecules Toward Specific Targets

As we know, the focus and key of the drug discovery process is to obtain novel molecules with good activity for the concerned biological target. So here, to evaluate the ability of our model to generate biologically active molecules, we used three popular targets including GLP‐1 receptor, HPK1, and LRRK2 as examples to analyze the binding affinity and binding pose of generated molecules.

Compared with molecular generation models, virtual screening (VS) is a commonly used computational technique to identify potential bioactive molecules by searching large compound libraries.^[^ ^37^ ^]^ However, the virtual screening methods generally only can identify lead compounds from the known and limited chemical space. Relatively, molecular generation methods excel in exploring novel chemical space and optimizing specific properties. Here, to verify if our molecular generation model can explore new chemical space and obtain structurally novel and active molecules, we utilized three popular targets GLP‐1 receptor, HPK1, and LRRK2 to compare the features of hit compounds by virtual screening and generated by our DiffMC‐Gen model. For virtual screening, a multi‐hierarchical workflow comprising HTVS, Glide‐SP, and Glide‐XP in Schrödinger was applied, retaining the top 10% of compounds at each stage.^[^ ^38^ ^]^ The top 1000 molecules for each target screened from the MOSES database were chosen to compare with the 1000 molecules generated by our DiffMC‐Gen model.

**Figure 4:**
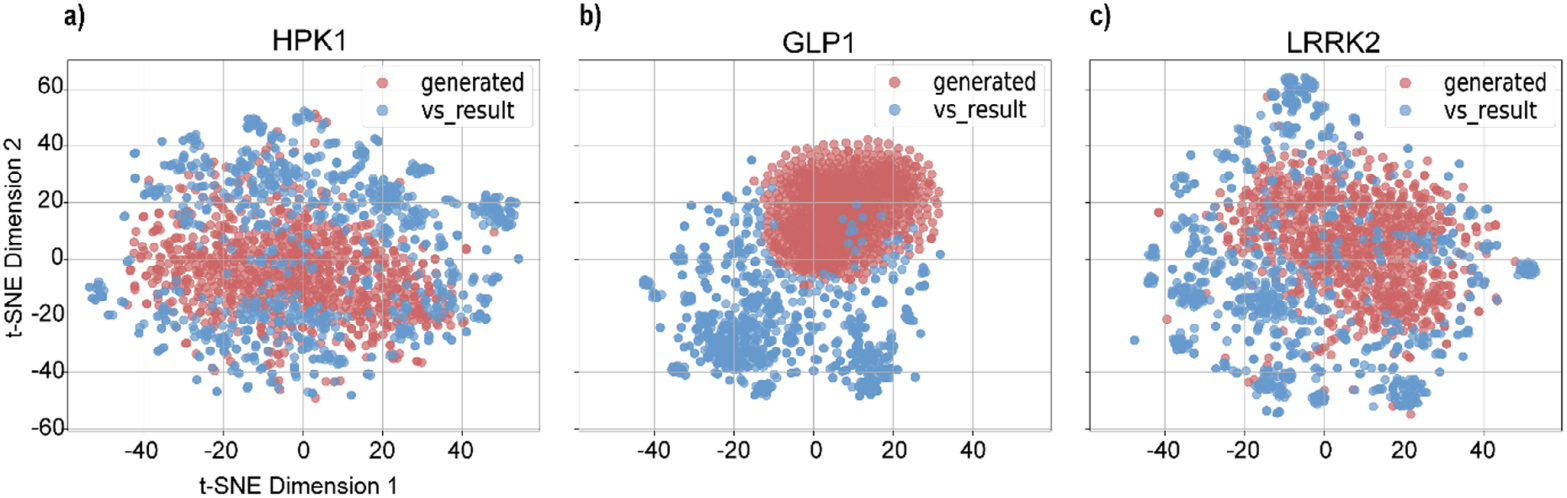
T‐SNE analysis of the chemical space of the VS results from the MOSES dataset and the molecules generated by DiffMC‐Gen (*n* = 1000).

T‐SNE (t‐distributed stochastic neighbor embedding) based on Extended‐Connectivity Fingerprints (ECFP) of molecules was applied to visualize the distribution of generated molecules and the molecules obtained by virtual screening.^[^ ^39^ ^]^ T‐SNE is a machine learning algorithm used for visualizing high‐dimensional data in a low‐dimensional space, typically 2D or 3D. The smaller the distance between data points, the higher the similarity between molecular structures. As shown in **Figure** **4**, the molecules generated by DiffMC‐Gen show different aggregation patterns with the molecules screened out from the MOSES database. The generated molecules targeting HPK1 and LRRK2 are favorably distributed in similar areas but tend to cluster in different regions compared to the screened molecules. This suggests that the generated molecules may share similar structural features with the screened molecules, indicating that our molecular generation model holds great promise for generating potentially active molecules during the exploration of new chemical space. The generated molecules targeting the GLP‐1 receptor exhibit a more distinct distribution from the screened molecules, indicating a certain degree of structural divergence relative to the generated molecules against two kinase targets. The difference in the distribution of chemical space between the generated molecules and the screened molecules can be attributed to the differences in the structure of the two types of targets themselves.

**Figure 5:**
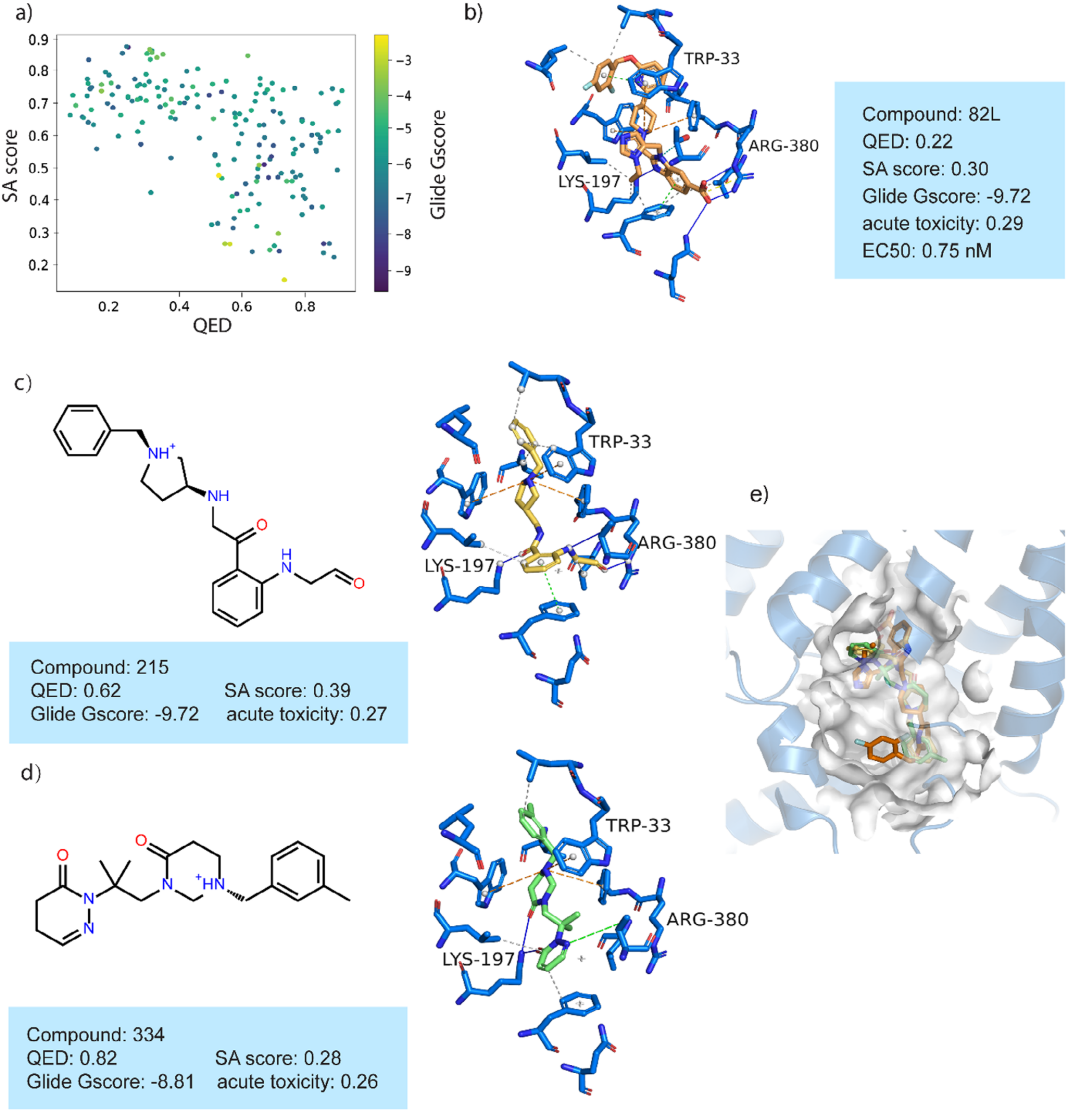
The distribution of properties of generated molecules and the binding pose of representative molecules targeting GLP‐1 receptor. a) The distribution of three key properties of generated molecules (*n* = 400). b) Interaction diagram between GLP‐1 receptor and one known agonist (PDB ID: 7S15). c, d) Two representative molecules and their detailed interactions with the GLP‐1 receptor. **e** Overlay of two representative molecules and known agonist in the binding site of GLP‐1 receptor.

The distribution of docking scores for the generated molecules, along with the binding mode analysis of representative molecules, can provide valuable insights into the potential of these molecules for target binding and therapeutic applications. Thus here, we first evaluated the performance of the generated molecules targeting the GLP‐1 receptor, a key target in treating type 2 diabetes and obesity by enhancing insulin secretion, reducing glucagon levels, and promoting satiety. **Figure** **5**a illustrates the distribution of three key properties in generated molecules, such as QED, SA score, and docking score to the target protein (PDB ID: 7S15). The aggregated region demonstrates that DiffMC‐Gen effectively generates molecules with favorable binding affinities and desirable physicochemical properties. Figure 5b illustrates the detailed interaction between one known agonist (EC_50_ = 0.75 nM) and the GLP‐1 receptor obtained from the PDB database (PDB ID: 7S15).^[^ ^40^ ^]^ Figure 5c,d shows the binding pose of representative molecules (compounds 215 and 334) generated by DiffMC‐Gen. These molecules form π‐cation interaction with Trp33 and strong hydrogen bonds with Lys197. In addition, compound 215 forms an ion‐pair interaction with Arg380. These generated molecules fit well within the binding pocket, sharing similar favorable interactions to target with known agonists. Figure 5e shows the overlay between two representative molecules and known agonists in the binding site of the GLP‐1 receptor. It can be seen from Figure 5e that two representative molecules share a similar binding mode with the known agonist, suggesting that small molecules generated by DiffMC‐Gen can form a favorable interaction with the GLP‐1 receptor and may produce the desired physiological response with high probability.

**Figure 6:**
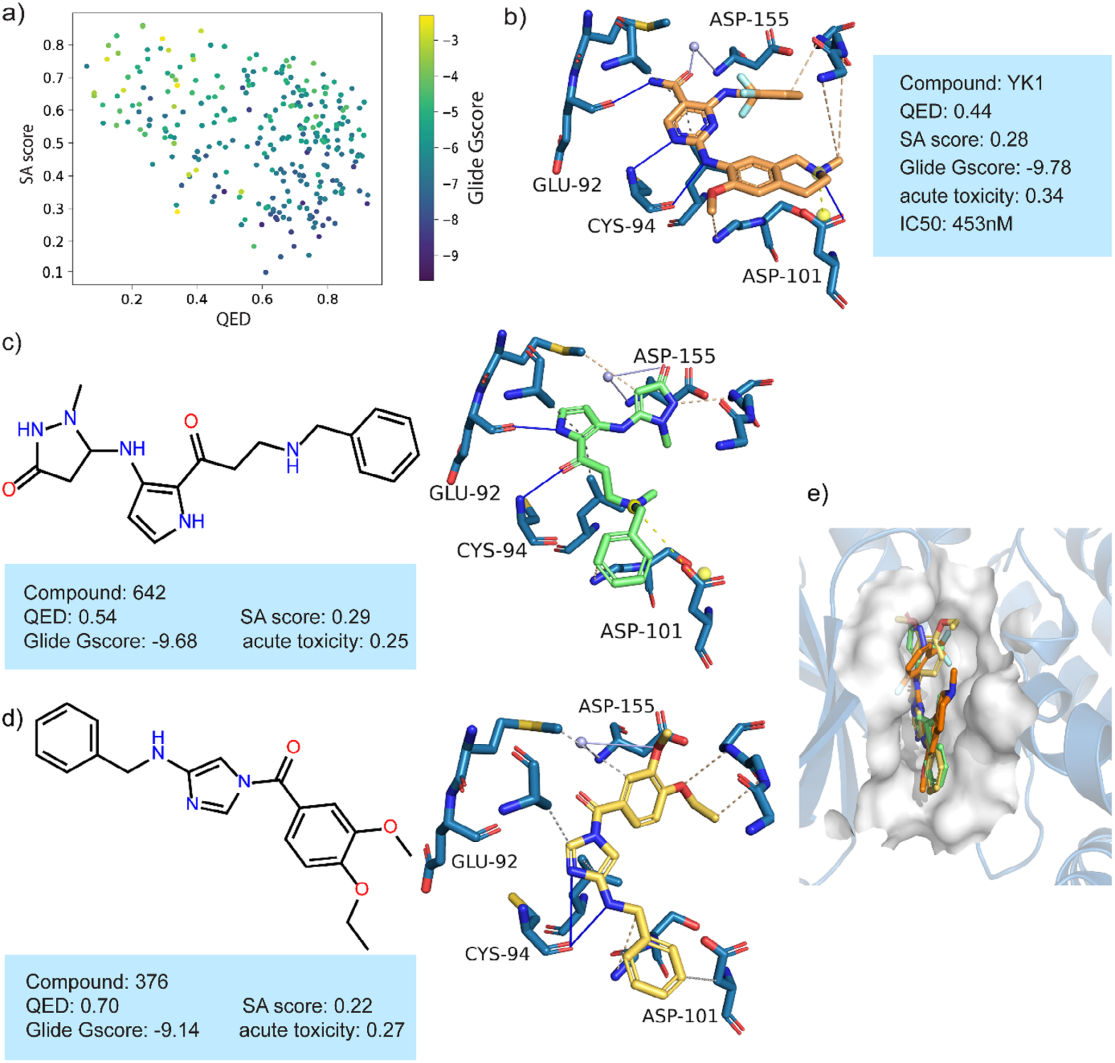
The distribution of properties of generated molecules and the binding pose of representative molecules targeting HPK1. a) The distribution of three key properties of generated molecules (*n* = 1000). b) Interaction diagram between HPK1 and one inhibitor (PDB ID: 7M0M). c, d) Two representative molecules and their detailed interactions with HPK1. e) Overlay of two representative molecules and known inhibitors in the binding site of HPK1.

**Figure** **6** shows the distribution of the docking score of generated molecules and the binding pose of known inhibitors and representative molecules targeting HPK1, which is a potential target for tumor immunotherapy and autoimmune diseases. Figure 6a presents the distribution of three critical properties for the molecules generated by DiffMC‐Gen. The clustering region of the three attributes is concentrated in the advantage range of each attribute, which indicates that the model has good structural optimization ability. The binding mode between one known inhibitor (IC_50 _= 453 nM) and HPK1 (PDB ID: 7M0M) is shown in Figure 6b.^[^ ^41^ ^]^ The binding poses of two represented generated molecules (compound 642 and 376) with top‐ranking docking scores are shown in Figure 6c,d. These generated molecules form similar hydrogen bond interaction with Cys94 and water‐mediated interaction with Asp155 to target. These interactions are critical for the activity and selectivity of HPK1. Besides, compound 642 forms a similar hydrogen bond interaction to Glu92 and a salt bridge to Asp101 as displayed in Figure 6b. These generated molecules share similar interactions to target with established inhibitors, demonstrating the capability of DiffMC‐Gen to design molecules with potential pharmacological activity. Moreover, Figure 6e shows the well‐aligned overlaps between generated molecules and known inhibitors in the binding site of HPK1, further validating the effectiveness of DiffMC‐Gen.

**Figure 7:**
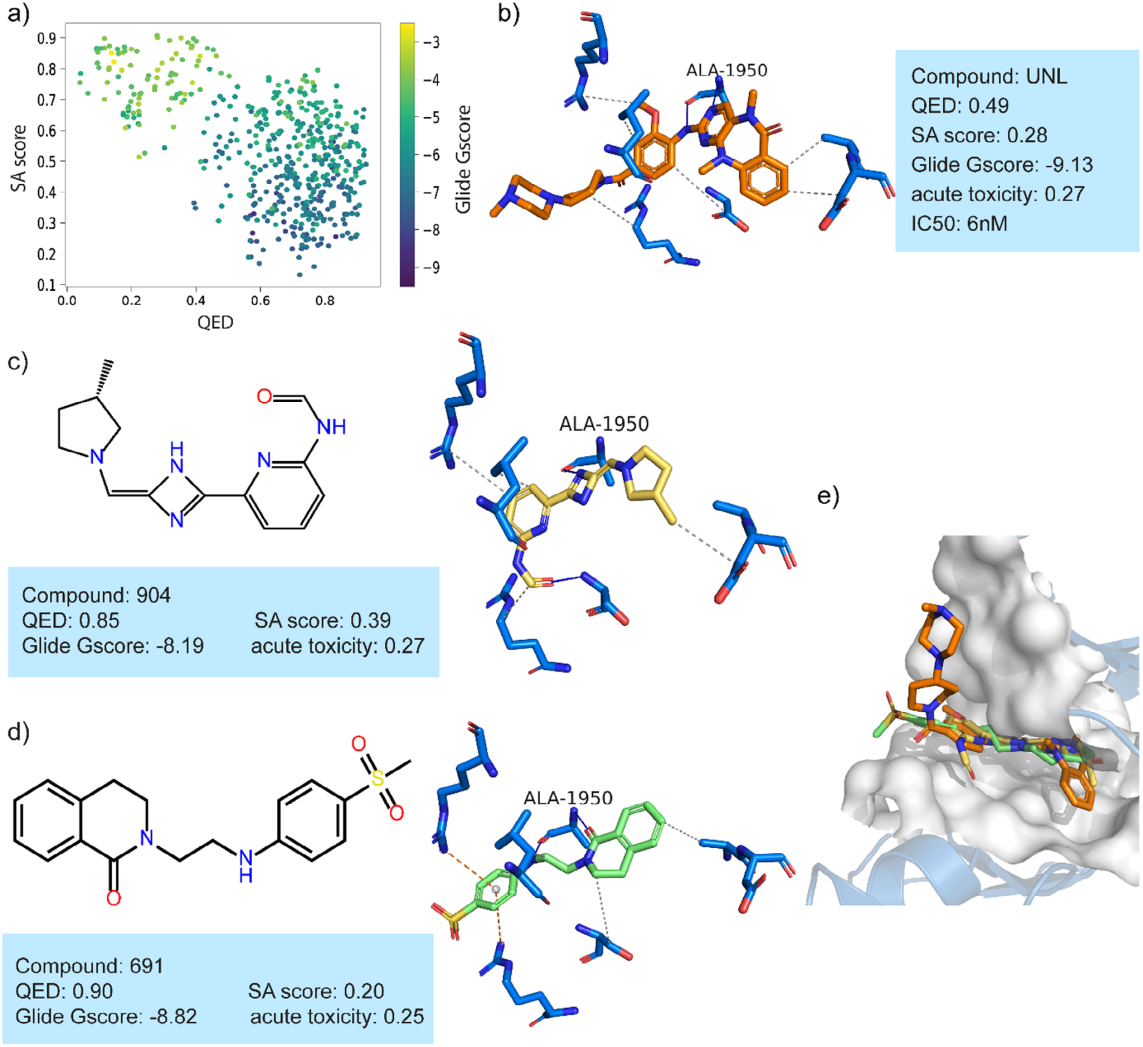
The distribution of properties of generated molecules and the binding pose of representative molecules targeting LRRK2. a) The distribution of three key properties of generated molecules (*n* = 1000). b) Interaction diagram between LRRK2 and one inhibitor (PDB ID: 8FO7). c, d) Two representative molecules and their detailed interactions with LRRK2. e) Overlay of two representative molecules and known inhibitors in the binding site of LRRK2.

We also assess the generation performance of a model targeting LRRK2, a kinase target for developing potential therapies for Parkinson's disease and other neurodegenerative disorders. From the distribution of the three key attributes, as depicted in **Figure** **7**a, DiffMC‐Gen can effectively generate molecules with good binding affinity and low synthetic complexity as well as high drug‐likeness estimates. The detailed interactions of a known inhibitor LRRK2‐IN‐1 in complex with LRRK2 (PDB ID: 8FO7) is shown in Figure 7b.^[^ ^42^ ^]^ Figure 7c,d shows the binding pose of two representative generated molecules (compound 904 and 691) with better docking results. These two molecules form hydrogen bonds to Ala1950 and van der Waals interactions with surrounding residues in the binding pocket. These interactions are similar to those between LRRK2 and known inhibitors, supporting their potential activity. Figure 7e presents the overlay of two representative molecules with LRRK2‐IN‐1 in the LRRK2 binding site. Both representative molecules adopt binding modes similar to that of the known inhibitor. The results indicate that our model can explore novel molecular structures with potential activity.

## 4 Conclusion

In this paper, we developed a novel molecular generation model DiffMC‐Gen for multi‐conditional molecular generation by integrating both discrete and continuous diffusion models to enhance its ability to perceive 3D molecular structures. In the discrete graph diffusion network, a dynamically composable multi‐head attention mechanism is employed, combining attention scores and conditional weight matrices to reduce computational cost while enhancing the influence of conditional information during diffusion steps. In the continuous graph diffusion network, not only a novel hierarchy of SE(3)‐equivariant local isomorphisms is used to evaluate local structural similarities but a local frame transition block is also used to capture global changes in local geometries. Additionally, it involves a multi‐objective optimization strategy to simultaneously optimize multiple properties of generated molecules, including binding affinity, drug‐likeness, synthesizability, and toxicity.

The comparison between DiffMC‐Gen and baseline models verifies that DiffMC‐Gen consistently outperforms or matches baseline models in both the general generation performance and the distribution of expected properties of generated molecules. Through the case studies using three targets—HPK1, LRRK2, and GLP‐1 receptor, the obtained results show that the generated molecules based on DiffMC‐Gen not only have good biological activity but also maintain good druglike properties, structural diversity, and novelty, highlighting that our method can generate high‐quality candidature with expected properties and will become a useful de novo drug design tool.

## Conflict of Interest

The authors declare no conflict of interest.

## Supporting information



Supporting Information

## Data Availability

The data that support the findings of this study are available from the corresponding author upon reasonable request.
