# Supplementary material for: DiffMC‐Gen: A Dual Denoising Diffusion Model for Multi‐Conditional Molecular Generation
Source: Adv Sci (Weinh). 2025 Apr 1;12(22):2417726. doi: 10.1002/advs.202417726 (PMC12165109; doi:10.1002/advs.202417726)
Supplement: Supplementary file 1 — Supporting Information [file ADVS-12-2417726-s001.docx]

Supporting Information

DiffMC-Gen: A Dual Denoising Diffusion Model for Multi-Conditional Molecular Generation

Yuwei Yang, Shukai Gu, Bo Liu, Xiaoqing Gong, Ruiqiang Lu, Jiayue Qiu, Xiaojun Yao*, Huanxiang Liu*

[1. Dataset Statistics 2](#_Toc192623090)

[1.1 Three training sets 2](#_Toc192623091)

[1.2 Target information 2](#_Toc192623092)

[1.3 Abbreviations 3](#_Toc192623093)

[2. Pharmacophore extracted process 4](#_Toc192623094)

[3. Ablation studies 5](#_Toc192623095)

[4. Assessment of Toxicity Prediction Models 6](#_Toc192623096)

[Reference 9](#_Toc192623097)

# 1. Dataset Statistics

## 1.1 Three training sets

The statistical analysis about the number and the structural information of contained compounds in three training set were performed and the detailed information was given in Table S1.

**Table S1**. Dataset information for all multi-conditional generation tasks.

| Datasets | Molecule  (Train/Validation/Test) | Heavy Atom Number | Min Atoms | Max Atoms | Avg. Atoms | Min Bonds | Max Bonds | Avg. Bonds |
| --- | --- | --- | --- | --- | --- | --- | --- | --- |
| Qm9 | 133885  (100000/20497/13388) | 6 | 3 | 29 | 18.27 | 2 | 28 | 18.86 |
| CSD | 173641  (110591/36864/36864) | 15 | 3 | 40 | 30.24 | 2 | 50 | 31.54 |
| MOSES | 1936962  (1584663/176074/176225) | 9 | 8 | 62 | 39.63 | 7 | 64 | 41.20 |

## 1.2 Target information

Three targets including HPK1, LRRK2 and GLP-1 receptor were used to evaluate our molecular generation model. The detailed medicinal therapeutic significance of three targets are described as below.

*HPK1 (Hematopoietic progenitor kinase 1)* also known as MAP4K1, is a hematopoietic cell-restricted member of the Ste20 family of serine/threonine kinases that functions as a negative regulator of activation signals generated by the T-cell receptor, as such is an attractive target for cancer immunotherapy.

*LRRK2 (Leucine-rich repeat kinase 2)* is a protein kinase associated with familial and sporadic Parkinson's disease, playing a crucial role in neuronal function and survival.

*GLP-1 (Glucagon-Like Peptide-1)* *receptor* is a hormone belonging to the incretin family, which helps to regulate glucose metabolism by enhancing insulin secretion and lowering blood sugar levels, is used therapeutically to manage type 2 diabetes and obesity.

## 1.3 Abbreviations

**Table S2** provides a comprehensive list of abbreviations and their corresponding full names used in the main text.

**Table S2.** Abbreviations and their full name.

| Abbreviation | Full Term |
| --- | --- |
| CADD | Computer Aided Drug Discovery |
| CCDC | Cambridge Crystallographic Data Centre |
| CSD | Cambridge Structural Database |
| DCMHA | Dynamically Composable Multi-Head Attention |
| DiffMC-Gen | Dual denoising diffusion model for Multi-Conditional molecular Generation |
| DiGress | Discrete Denoising diffusion models for graph generation |
| ECFP | Extended-Connectivity Fingerprints |
| EGNN | Equivariant Graph Neural Network |
| FCD | the Fréchet ChemNet Distance between the distributions of generated and real molecules |
| GCDM | Geometry-Complete Diffusion for 3D Molecule Generation and Optimization |
| GCPR | G protein-coupled receptor |
| GDSS | Graph Diffusion via the System of SDEs |
| GeoLDM | Geometric Latent Diffusion Models |
| GFMDiff | Geometric-Facilitated Denoising Diffusion Model for 3D Molecule Generation |
| GLP-1 | Glucagon-Like Peptide-1 |
| GNN | Graph neural network |
| Graph DiT | Graph Diffusion Transformer for Multi-Conditional Molecular Generation |
| Gscore | Glide score estimates the binding affinity between ligand and target, calculated by Glide SP from Schrödinger software. |
| HPK1 | Hematopoietic progenitor kinase 1 |
| IntDiv | Internal Diversity within the generated set |
| JTVAE-BO | Junction Tree Variational Autoencoder with Bayesian Optimization |
| LD50 | Lethal Dose Fifty |
| LRRK2 | Leucine-rich repeat kinase 2 |
| LSTM-HC | Long Short Term Memory on SMILES with Hill Climbing |
| MARS | Markov Molecular Sampling for Multi-objective Drug Discovery |
| MDM | Molecular Diffusion Model for 3D Molecule Generation |
| MMFF94 | Merck Molecular Force Field |
| MOSES | Molecular Sets |
| NLP | Natural Language Processing |
| PEGNN | Powerful and Efficient geometric Graph Neural Networks |
| QED | Quantitative Estimate of Drug-likeness |
| QM9 | Quantum Machine 9 |
| SA score | Synthetic Accessibility score |
| SDEs | Stochastic Differential Equations |
| SOTA | State Of The Art |
| t-SNE | t-distributed Stochastic Neighbor Embedding |
| UFF | Universal Force Field |
| VS | Virtual Screening |

# 2. Pharmacophore extracted process

In this work, ligand-based pharmacophore model was constructed and used as one constraint of our molecular generation model. The detailed methods and process to construct pharmacophore model were described as below. We downloaded the related inhibitors or agonists from the PubChem. After calculating Extended-Connectivity Fingerprints (ECFPs) of their scaffolds, the selected active small molecules of each target can be categorized into five distinct classes by K-Nearest Neighbor (KNN) algorithm. Common functional moieties were identified within each class, and these features were utilized to construct a pharmacophore model for each target. The spatial distribution of pharmacophore sites and the pharmacophoric features of reference dataset are represented as complete graph based on bonding pathways. Then the graph matching score to the pharmacophore is constrained as the highest one of attributes. The pharmacophore matching score ranges from 0 (unfavorable) to 1 (favorable).


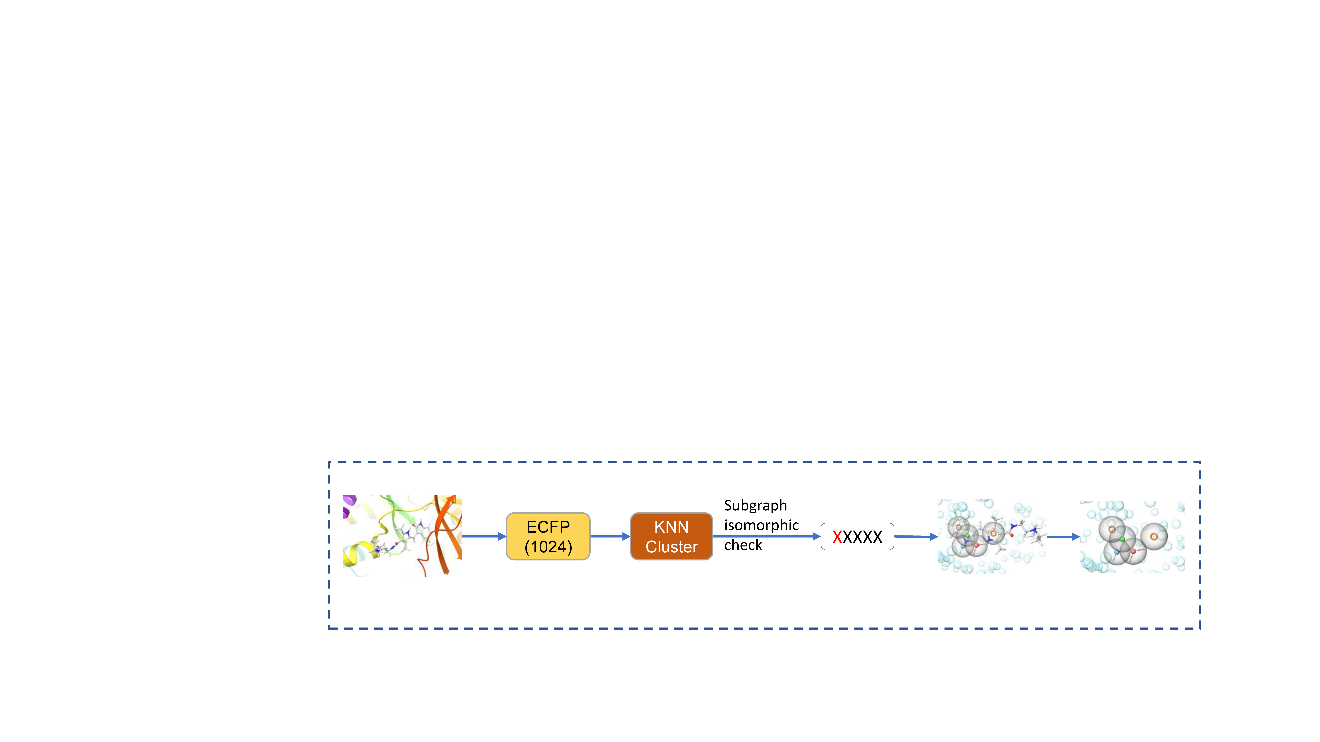


**Figure S1.** The pharmacophore extracted process.

# 3. Ablation studies

The design of DiffMC-Gen is guided by the goal of generating small molecules with expected properties by multi-objective optimization strategy. Therefore, to analyze the impact of key components in DiffMC-Gen, we conducted a series of ablation experiments. Several evaluation metrics including validity, uniqueness, novelty and four optimized properties are used to assess the performance of each module. The molecules in the CSD dataset exhibit geometries that closely reflect real-world structures, making it an ideal choice to evaluate the performance of generation model to produce realistic molecular conformations. In the ablation study, molecular generation was performed by using LRRK2 as target.

As shown in **Table S3**, the baseline model, which employs a simple combination of a Graph Transformer and EGNN, provides a fundamental representation of molecular structures but lacks sufficient coordination between local and global features, leading to suboptimal validity. Replacing the original Graph Transformer with an updated version that incorporates Dynamically Composable Multi-Head Attention (DCMHA) mechanism improves molecular representation learning, resulting in better target-conditioned guidance. However, conditional EGNN improves pharmacophore matching ability twice but increases structural complexity. Such dual denoising networks cannot adequately balance the guidance of multiple attributes and local structural features well. Furthermore, a powerful and efficient geometric graph neural network (PEGNN) is integrated that refines spatial relationship modeling, thereby improving the model’s ability of capturing three-dimensional structural constraints. This new combination balances the multiple properties within the dual denoising process, especially enhancing the generation of chemically meaningful and structurally diverse molecules. To develop a model that balances molecular quality, diversity, and drug-likeness, it is essential to integrate a multi-objective optimization strategy along with joint training of the unconditional model. Comparative results suggest that this approach further refines the molecular generation process and significantly enhances the validity of the generated molecules. These results underscore the necessity of progressively refining both the graph-based and geometric components while incorporating chemical-relevant optimization strategies to achieve optimal molecular generation performance.

**Table S3**. Ablation experimental results of DiffMC-Gen’s components (n=1000).

| Denoising network | Validity | Novelty | Uniqueness | Pharmacophore matching score | QED | SA score | Acute toxicity |
| --- | --- | --- | --- | --- | --- | --- | --- |
| Graph Trans ^a)^ +EGNN | 0.21 | 0.98 | 0.99 | 0.46 | 0.48 | 0.41 | **0.23** |
| DCMHA Trans ^b)^ + EGNN | 0.22 | 1.00 | 1.00 | 0.69 | 0.37 | 0.72 | 0.35 |
| DCMHA Trans + PEGNN ^c)^ | 0.25 | 1.00 | 1.00 | 0.54 | 0.60 | 0.55 | 0.25 |
| **DCMHA Trans + PEGNN**  **(multi-objective optimization)^d)^** | **0.75** | 0.99 | 0.99 | **0.69** | **0.71** | **0.39** | 0.25 |

^a)^ Graph Trans means Graph transformer framework; ^b)^ DCMHA Trans means Graph transformer combined with Dynamically Composable Multi-Head Attention mechanism (DCMHA), which is used as discrete denoising network in DiffMC-Gen; ^c)^ PEGNN means powerful and efficient geometric graph neural networks, which is used as continous denoising network in DiffMC-Gen; ^d)^ This is the final framework of DiffMC-Gen.

# 4. Assessment of Toxicity Prediction Models

We built a comprehensive predictive model for integrated Gradient Boosted Regression (GBR), Support Vector Regression (SVR), and K -nearest Neighbors regressor (KNN) using machine learning, combining an integrated learning strategy with Extreme Gradient Boosting Regression (XGB-R) as an integrated predictor. The GBR model is a boosting technique that sequentially builds an ensemble of weak learners, typically decision trees. SVR uses kernel function that maps the input features into a higher-dimensional space to find linear separation between the independent and dependent variables. KNN is a simple and intuitive algorithm that makes predictions by finding the K nearest data points to a given input and averaging their target values. Choosing the right value for K and the appropriate distance metric can impact the quality of the predictions. All ML algorithms first use Grid search cross-validation to produce the optimal hyper-parameters for the corresponding model. The relationship between them is shown in **Figure S2**. We trained GBR, SVR, and KNN in the Oral Acute Toxicity Data Set in rats, respectively, and there is room for optimization of test results as in **Figure S3**. Integrated learning strategies improve learner prediction. XGB-R builds an ensemble of above weak learners, it produces models in a step-by-step fashion, with every new model aiming to fix the mistakes of the preceding models. As the prediction distribution, there is a certain improvement in the forecast effect after integration, which is more closely aligned with the desired curve.


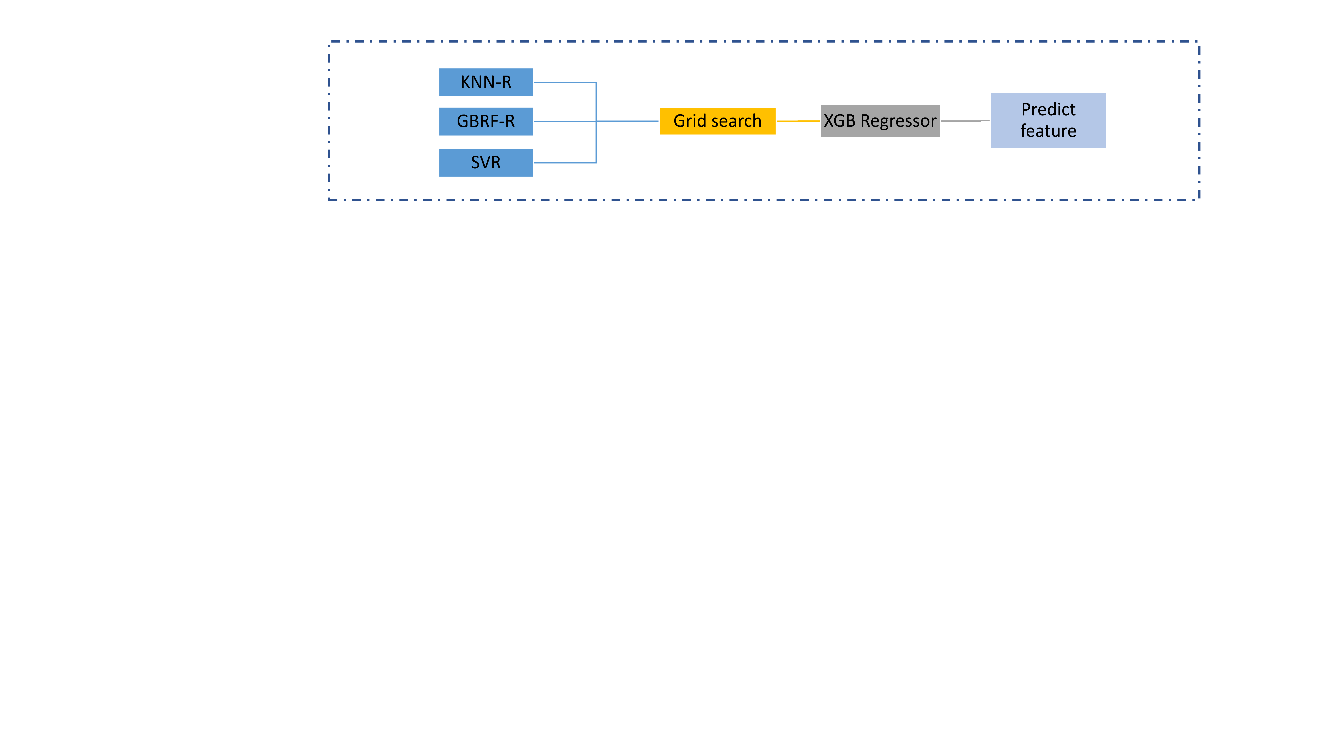


**Figure S2.** The pipeline of acute toxicity prediction.


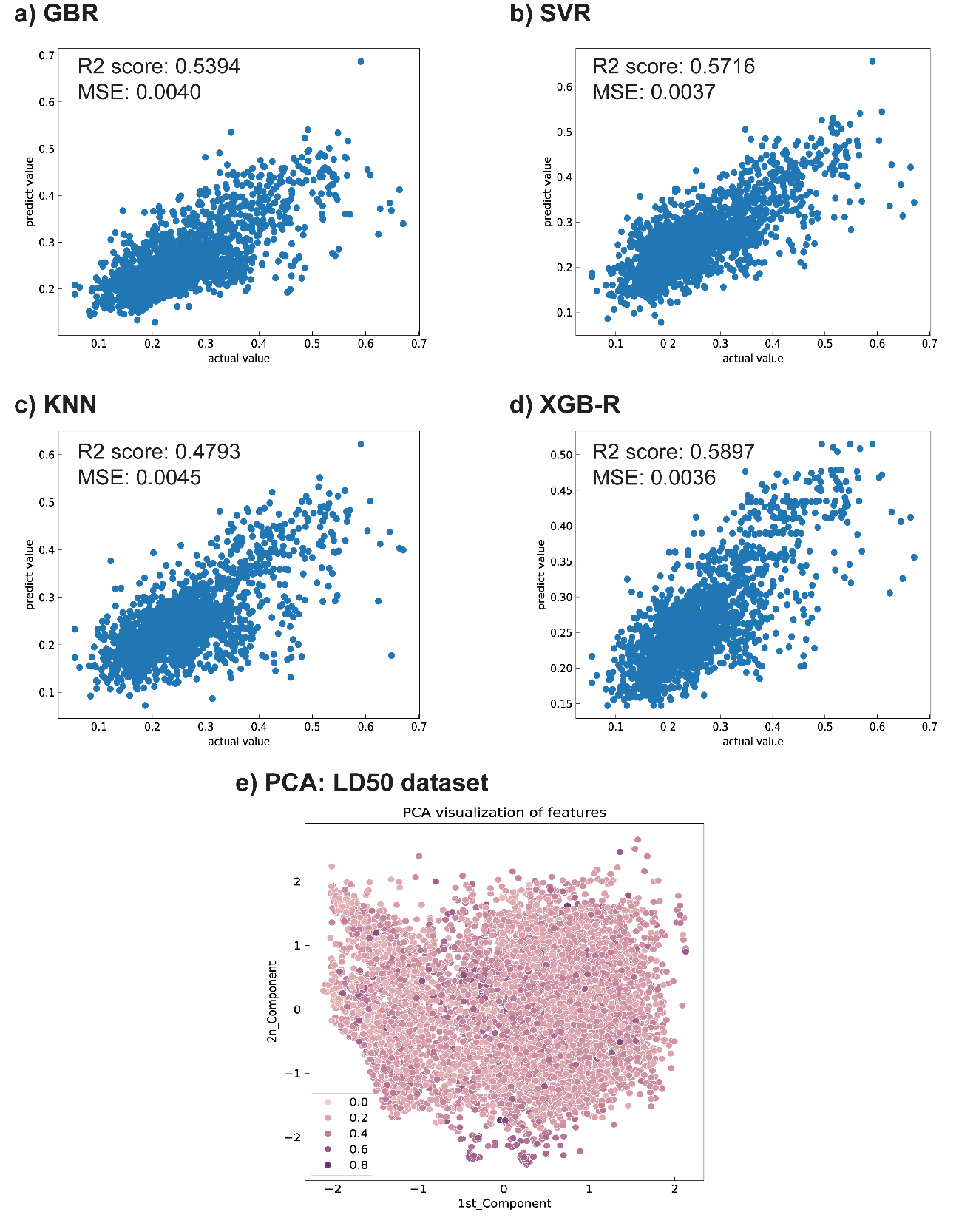


**Figure S3.** Analysis results of the acute toxicity score prediction. The four components **a)** Gradient Boosting Random Forest Regressor (GBR)**,** **b)** Epsilon-Support Vector Regressor (SVR), **c)** Regression based on k-nearest neighbors (KNN), and **d)** ensembled XGBoost Regressor (XGB-R) of predictive model present optimal performance of parametric grid search post in acute toxicity datasets (Rat LD50 Oral). **e)** The PCA visualization of training dataset of predictor indicates the uniform value distribution.

# Reference

[1] a) B. J. Bender, S. Gahbauer, A. Luttens, J. Lyu, C. M. Webb, R. M. Stein, E. A. Fink, T. E. Balius, J. Carlsson, J. J. Irwin, B. K. Shoichet, *Nat. Protoc.* **2021**, *16* (10), 4799; b) Z. Wang, H. Sun, X. Yao, D. Li, L. Xu, Y. Li, S. Tian, T. Hou, *Phys. Chem. Chem. Phys.* **2016**, *18* (18), 12964.
